# Urinary cell-free mitochondrial and nuclear deoxyribonucleic acid correlates with the prognosis of chronic kidney diseases

**DOI:** 10.1186/s12882-019-1549-x

**Published:** 2019-10-28

**Authors:** Chia-Chu Chang, Ping-Fang Chiu, Chia-Lin Wu, Cheng-Ling Kuo, Ching-Shan Huang, Chin-San Liu, Ching-Hui Huang

**Affiliations:** 1 Department of Internal Medicine, Kuang Tien General Hospital, Taichung, Taiwan; 2 Department of Nutrition, Hungkuang University, Taichung, Taiwan; 3 School of Medicine, Chung Shan Medical University, Taichung, Taiwan; 4 Nephrology Division, Department of Internal Medicine, Changhua Christian Hospital, Changhua, Taiwan; 5 Vascular & Genomic Research Center, Changhua Christian Hospital, Changhua, Taiwan; 6 Center of General Education Tunghai University, Taichung, Taiwan; 7 Internal Medicine Research Center, Changhua Christian Hospital, Changhua, Taiwan; 8 Department of Neurology, Changhua Christian Hospital, Changhua, Taiwan; 9 Department of Cardiology, Changhua Christian Hospital, Changhua, Taiwan; 10 Institute of Statistics and Information Science, National Changhua University of Education, Changhua, Taiwan; 11 School of Medicine, College of Medicine, Kaohsiung Medical University, Kaohsiung, Taiwan; 12 Department of Beauty Science and Graduate Institute of Beauty Science Technology, Chienkuo Technology University, Changhua, Taiwan

**Keywords:** Cell-free mitochondrial deoxyribonucleic acid, Cf-mtDNA, Cell-free nuclear deoxyribonucleic acid, Cf-nDNA, Neutrophil gelatinase-associated lipocalin, NGAL, Chronic kidney disease

## Abstract

**Introduction:**

Cell-free deoxyribonucleic acid DNA (cf-DNA) in urine is promising due to the advantage of urine as an easily obtained and non-invasive sample source over tissue and blood. In clinical practice, it is important to identify non-invasive biomarkers of chronic kidney disease (CKD) in monitoring and surveillance of disease progression. Information is limited, however, regarding the relationship between urine and plasma cf-DNA and the renal outcome in CKD patients.

**Methods:**

One hundred and thirty-one CKD patients were enrolled between January 2016 and September 2018. Baseline urine and plasma cell-free mitochondrial DNA (cf-mtDNA) and cell-free nuclear DNA (cf-nDNA) were isolated using quantitative real-time PCR. Estimated glomerular filtration rate (eGFR) measurement was performed at baseline and 6-month follow-up. Favorable renal outcome was defined as eGFR at 6 months minus baseline eGFR> = 0. Receiver operator characteristics (ROC) curve analysis was performed to assess different samples of cf-DNA to predict favorable renal outcomes at 6 months. A multivariate linear regression model was used to evaluate independent associations between possible predictors and different samples of cf-DNA.

**Results:**

Patients with an advanced stage of CKD has significantly low plasma cf-nDNA and high plasma neutrophil gelatinase-associated lipocalin (NGAL) levels. Low urine cf-mtDNA, cf-nDNA levels and low plasma NGAL were significantly correlated with favorable renal outcomes at 6 months. The urine albumin-creatinine ratio (ACR) or urine protein-creatinine ratio (PCR) level is a robust predictor of cf-mtDNA and cf-nDNA in CKD patients. Baseline urine levels of cf-mtDNA and cf-nDNA could predict renal outcomes at 6 months.

**Conclusions:**

Urinary cf-mtDNA and cf-nDNA may provide novel prognostic biomarkers for renal outcome in CKD patients. The levels of plasma cf-nDNA and plasma NGAL are significantly correlated with the severity of CKD.

**Electronic supplementary material:**

The online version of this article (10.1186/s12882-019-1549-x) contains supplementary material, which is available to authorized users.

## Background

Chronic kidney disease (CKD) is a global public health problem affecting up to 10% of the population worldwide [1], although guidelines for the clinical staging of CKD have been established [2]. The natural history of the earlier phases of CKD are highly unpredictable and the early identification of CKD and timely detection of progression are truly global challenges [3, 4]. It is important to recognize the risk factors of CKD, and better approaches for the prevention, early detection, and treatment of CKD [5].

The kidney is a highly energetic organ and rich in mitochondria and numerous studies have shown that mitochondrial dysfunction contributes to different types of kidney diseases, including diabetic and nondiabetic nephropathy [6]. Proteinuria, caused by a primary insult to the kidney, induces oxidative stress in renal tubular cells and causes mitochondrial dysfunction [7, 8], which leads to cellular damage by reactive oxygen species generation as well as epithelial–mesenchymal transition (EMT) [9, 10]. To date, CKD is characterized by mitochondrial dysfunction, oxidative stress, and aberrant autophagy [11, 12] in addition to significant changes in the activation of transforming growth factor-β, p53, hypoxia-inducible factor, chronic inflammation, and traditional vascular dysfunction [11]. By the experimental models of CKD, preventing mitochondrial dysfunction inhibits renal tubular cell EMT and renal fibrosis [13]. However, only a relatively small number of translational studies have shown the clinical relevance of these mechanisms between renal diseases and mitochondrial dysfunction in humans [6, 14, 15].

Free circulating nucleic acids, cell-free deoxyribonucleic acid (cf-DNA), were discovered in human plasma in 1948 by Mandel and Metais [16]. Some studies showed the damaged mitochondria release their DNA content into the systemic circulation [17, 18]. Thus, cell-free mitochondrial deoxyribonucleic acid (cf-mtDNA) is easily detected in plasma, and has been explored as a biomarker of various diseases [19–21]. Recently, increased plasma levels of cf-mtDNA have been reported to correlate with the severity of injury in patients sustaining polytrauma [22] and the severity of stroke [23], be a prognostic marker of acute myocardial infarction [24] and intensive care unit patients [25, 26]. Alterations of cf-mtDNA in the blood also might be associated with several systemic diseases, including primary mitochondrial disorders, carcinogenesis, and hematologic diseases [27, 28]. In a community-based population cohort, higher plasma cf-mtDNA was associated with a lower incidence of CKD independent of traditional risk factors [20] and was associated with a lower prevalence of microalbuminuria [29]. Apart from blood, cf-DNA could also be detected in urine. Urine cf-DNA originates either from cells shedding into urine from genitourinary tract, or from cell free DNA in circulation passing through glomerular filtration [30]. The presence of genetic information in urine has been observed in some clinical studies. For examples, the urinary cf-mtDNA level had statistically significant correlations with the peak serum creatinine level and the duration of hospitalization in a study of acute kidney injury (AKI) [30]. Patients who required temporary dialysis also tended to have higher urinary cf-mtDNA levels than those without dialysis [31], but no relationship between the urinary cf-mtDNA level and renal outcomes has been reported. A recent study showed that urinary cf-mtDNA level was increased in mice after 10–15 min of ischemia, and that the level correlated with the duration of ischemia [32]. Otherwise, platelet and leukocyte counts in samples are important sources of variation when cf-DNA is measured in DNA extracted from whole blood [27, 28]. Emerging data showed that measuring cf-DNA extracted from whole blood (nuclear deoxyribonucleic acid, cf-nDNA) could yield different results from peripheral blood mononuclear cells or buffy coat (cf-mtDNA), because of the presence of mitochondrial DNA in platelets [27, 28].

Since the majority of urine cf-DNA originates from apoptosis or necrosis of cells exfoliated from urogenital system [30]. And the minority are originating from blood circulation, which contains important genomic information from various positions all over the body [33, 34]. We postulate that urine cf-DNA might be regarded as a marker which combined genetic information from urogenital system and systemic. We hypothesis that urinary cf-DNA might be a better novel biomarker than circulating cf-DNA in predicting renal outcome in CKD patients. To clarify the clinical application of cf-mtDNA and cf-nDNA in CKD, we studied the correlation between cf-mtDNA and cf-nDNA, both urine and plasma, and the stage of CKD or prognosis of renal outcomes.

## Methods

The Institutional Review Board of Changhua Christian Hospital approved the experimental protocols (approval number 140306) and all the participants provided written informed consent to participate the study. All patients of the study joined our nationwide preventive multidisciplinary program, also regulated by the Clinical Care Program Certification and Joint Commission International, for early CKD or pre-ESRD (end stage renal disease). We investigated patients who were enrolled in our CKD care program between January 2016 and September 2018.The goals for patients’ blood pressure, glucose and lipid control were based on the KDOGI guidelines.

Overall, of 131 patients with CKD, 7 patients dropped-out for the duration of follow-up less than 6 months, and 52 volunteers were recruited from the Nephrology Clinic at Changhua Christian Hospital, a tertiary referral hospital in Taiwan. The duration of follow-up for CKD was more than 6 months in all patients. The patients with following criteria were excluded: infection, acute fever, hepatic or cardiac disease, endocrinopathy, glomerulonephritis proved by biopsy or treatment with steroids or immunosuppressants, surgery, trauma, missing data at baseline, prior kidney transplant, acute kidney injury and a history of RRT or hospitalization for any cause in the past 3 months. The amount of proteinuria was calculated by urinary protein-creatinine ratio (PCR, mg/g) or albumin-creatinine ratio (ACR, mg/g). Microalbuminuria was established when two out of three ACR determinations were found to be within the range of 30–300 mg/g in a 6-month period. We calculated the glomerular filtration rate (eGFR) of the patients according to the CKD Epidemiology Collaboration equation (eGFR_CKD-EPI_) [35]. The stages of CKD were defined as follows: stage 1, eGFR > 90 ml/min/1.73 m^2^; stage 2, eGFR 60–89 ml/min/1.73 m^2^; stage 3a, eGFR 45–59 ml/min/1.73 m^2^; stage 3b, eGFR 30–44 ml/min/1.73 m^2^; stage 4, eGFR 15–29 ml/min/1.73 m^2^ and stage 5, eGFR < 15 ml/min/1.73 m^2^ or maintenance on RRT. We divided the population to early CKD (stages 1–3a) and advanced CKD (stages 3b–5) in the study, based on our national CKD care program.

Studies in CKD always address primary outcomes of death, ESRD, or doubling of baseline serum creatinine. Coresh et al. extended use of percentage reduction in eGFR as a surrogate for hard outcomes and they reported that longer–term follow-up, more than 1 year, was strongly predictive of ESRD and death [36]. We, therefore, examined the change of eGFR as a surrogate for hard outcomes within 6 months. For study simplicity, favorable renal outcome was defined as eGFR at 6 months minus baseline eGFR> = 0; unfavorable renal outcome indicated eGFR at 6 months minus baseline eGFR <0.

### Blood, plasma and urine sampling

Participants’ venous blood samples, following 8-h overnight fasting, were obtained and first morning urine samples were collected. Aliquots of urine were immediately frozen at − 80 °C until further analysis, but specimen reserved for no longer than 1 month. All assays were undertaken in duplicate with intra-assay variation coefficient less than 5%. Urinary albumin concentration was measured by an immunoturbidimetric method (Roche Diagnostics GmbH, Mannheim, Germany).

### DNA isolation and qPCR

The baseline data of urine and plasma cell-free mitochondrial DNA (cf-mtDNA) and cell-free nuclear DNA (cf-nDNA) were isolated from 131 patients with CKD in our study. Blood collection were drawn from each subject in the morning after overnight fasting 8 h. For each subject, 5 ml of whole blood was withdrawn from an antecubital vein and quickly delivered into an EDTA-K3-containing plastic tube. Plasma was collected by centrifugation of blood at 2500 rpm for 10 min, divided into several aliquots, and stored in − 80 °C until analysis. Urine samples were placed on 4 °C, centrifuged at 12000 rpm for 15 min within 8 h of collection, and the urine supernatants were separated and stored at − 80 °C until extraction DNA. Plasma and Urine cfDNA was extracted urine the Viral DNA mini Kit (AccuBioMed, Co, Ltd., Taiwan), following the protocol of manufacturer.

### Leukocyte mitochondrial DNA copy number

We used a LightCycler® 480 Instrument (Roche, Mannheim, Germany) to measure mitochondrial copy number (MCN) in leukocytes. Briefly, we extracted total DNA using the Gentra Puregene DNA kit (Qiagen, Hilden, Germany). Real-time polymerase chain reaction (PCR) was used to amplify the ND1 gene of mtDNA and β-globin of nuclear DNA, respectively. The relative MCN of mtDNA was normalized to the β-globin gene.

### Quantification of urine and plasma cell-free DNA by real-time PCR

Baseline urine and plasma cf-mtDNA and cf-nDNA were quantified by real-time PCR using the LightCycler® 480 Instrument (Roche, Mannheim, Germany) using specific primers to amplify the β-globin (forward: 5′-GTG CAC CTG ACT CCT GAG GAG A-3′, reverse: 5′-CCT TGA TAC CAA CCT GCC CAG-3′) and MT-ND1 (forward: AACATACCCATGGCCAACCT, reverse: AGCGAAGGGTTGTAGTAGCCC) genes from urine and plasma DNA, and standard regression analyses were used to derive the amount of urine and plasma nuclear DNA and mitochondrial DNA. The DNA concentrations were expressed as genome equivalents (GE)/mL of urine and plasma, where 1 GE was equivalent to 6.6 ng DNA [37]. Serially diluted human genomic DNA solution were used for preparing a six-point calibration curve [38].

### Plasma neutrophil gelatinase-associated lipocalin (NGAL)

Plasma NGAL concentrations were measured using a commercially available assay kit (Immunology Consultants Laboratory, Inc., Oregon, USA).

### Statistical analysis

Results are presented as the median (interquartile range) or number (proportion, %). All data were analyzed by the ANOVA test, if p with significant will receive further post hoc test analysis. A multivariate linear regression model was used to evaluate independent associations between possible predictors and cf-mtDNA or cf-nDNA. Receiver operator characteristics (ROC) curve analysis was performed to assess different samples of cf-DNA and NGAL to predict renal disease related outcomes. Youden’s index is the sum of sensitivity and specificity minus one, which is the most commonly used criterion for cut-off point selection in the context of ROC curve analysis. The *maximum* value of the index may be used as a criterion for selecting the optimum cut-off point. We used logistic regression to analyze the significant predictors of dissimilar renal outcome at 6 months. Statistical methods were employed to determine the appropriate number of patient and control samples required to ensure meaningful and statistically significant data (with a power of 80%, a sample size of 38 was sufficient to detect an observed difference). All statistical analyses were performed using IBM SPSS 20 (SPSS, Inc., Chicago, IL, USA) and a qualified statistician was employed to determine which tests should be used and whether they performed the analysis. In all analyses, *P*-values of < 0.05 were considered statistically significant.

## Results

### The demographic and clinical characteristics of the varying stages of chronic kidney diseases

Table 1 shows significant differences in age, renal functions, phosphate and low-density lipoprotein levels among varying stages of chronic kidney diseases and the further post hoc test analysis showed CKD stage related. High incidence, 37 to 59%, of hypertension encounters in varying stage CKD patients. Concerning DM, the incidence is increasing comparable to CKD stage worsen, 12% DM in early stage CKD and 54% in stage 5 CKD.

**Table 1:** Demographic characteristics of varying stages of chronic kidney disease

Variables	Stage 1,2^a^	Stage 3A^b^	Stage 3B^c^	Stage 4,5^d^	*p* value	Post hoc
	*n* = 23	*n* = 28	*n* = 31	*n* = 42		
Age (year)	47.1 ± 12.7	53.6 ± 9.4	54.9 ± 10.5	58.7 ± 6.1	0.002*	a < c,d
Gender (M/F)	3/20	8/20	22/9	29/13	0.212	
Hypertension (%)	42%	37%	59%	54%	0.191	
Diabetes mellitus (%)	12%	11%	23%	54%	0.160	
Systolic blood pressure (mm Hg)	138 ± 18	144 ± 28	138 ± 24	142 ± 15	0.943	
Diastolic blood pressure (mm Hg)	90 ± 15	91 ± 4	84 ± 18	84 ± 12	0.784	
BMI (kg/m^2^)	27.8 ± 5.4	28.2 ± 6.1	26.2 ± 6.1	24.8 ± 4.3	0.172	
BUN (mg/dL)	14.3 ± 4.4	17.7 ± 3.8	31.4 ± 13.0	64.5 ± 27.1	<0.001**	a,b < c < d
eGFR (ml/min/1.73 m^2)	84.4 ± 20.2	52.1 ± 4.4	31 ± 9.6	11.5 ± 7.3	<0.001**	a > b > c > d
Phosphate (mg/dL)	3.5 ± 0.4	3.5 ± 0.6	4.0 ± 0.7	5.0 ± 1.1	<0.001**	a,b,c < d
Cholesterol (mg/dL)	198.3 ± 50.4	171.5 ± 31.8	196.6 ± 47.3	153.3 ± 44.5	0.001**	a,c > d
HDL-C (mg/dL)	47.8 ± 14.3	43.3 ± 9.8	46.1 ± 12.6	46.7 ± 21.2	0.677	
LDL-C (mg/dL)	118.2 ± 45.3	104.6 ± 31.4	114.9 ± 41.3	80.8 ± 26.7	0.040*	c > d
Fasting Glucose (mg/dL)	118.3 ± 46.1	106 ± 21.8	112.6 ± 43.5	94.9 ± 24.6	0.263	
HbA1C (%)	6.3 ± 1.4	6.0 ± 0.6	6.2 ± 1.1	6.1 ± 0.9	0.778	
Urine A/C ratio (mg/g)	438.0 ± 826.4	535.7 ± 1018.4	2121.3 ± 2540.7	1548.0 ± 2377.2	0.058	
WBC (×10^3^/μL)	7.1 ± 2.4	6.2 ± 1.9	6.4 ± 2.1	6.8 ± 2.0	0.323	
Uric Acid (mg/dL)	7.1 ± 1.2	7.0 ± 1.4	7.5 ± 2.6	7.0 ± 1.2	0.629	
Platelet(×10^3^/μL)	230.2 ± 72.6	209.2 ± 59.5	201.9 ± 51.1	195.1 ± 58.2	0.159	

*P*-value by One-Way ANOVA follow with Bonferroni multiple comparisons at type I error of 0.05 level

*P <0.05, **P <0.01. Gender (M/F), female/male; BMI, body mass index; BUN, blood urea nitrogen; HDL, high-density lipoprotein; LDL, low-density lipoprotein; HbA1C, glycated hemoglobin; WBC, white blood count; eGFR, estimated glomerular filtration rate; urine A/C ratio, urine albumin-creatinine ratio

Post hoc: ^a^indicates group stage 1,2; ^b^indicates group stage 3a; ^c^indicates group stage 3b; ^d^indicates group stage 4,5

### The levels of cell-free mitochondrial (cf-mtDNA) and nuclear deoxyribonucleic acid (cf-nDNA), both urine and plasma, in varying stages of CKD patients

The plasma levels of neutrophil gelatinase-associated lipocalin (NGAL) significantly increase among the advanced stages of CKD (Table 2, Additional file 1: Fig. S1A). The plasma levels of cf-nDNA decrease as the renal function deterioration (Additional file 1: Figure S1B), in contrast to cf-mtDNA and mitochondrial copy number (MCN), which is not significantly changed among varying stages of CKD patients. There was no significant changes in urinary cf-mtDNA and cf-nDNA levels for varying stages of CKD. Urinary 8-hydroxy- 2-deoxyguanosine (8-OHdG), a symbolic marker of oxidative stress, is also not significantly changed in our study. In post hoc analysis, the data reveals that base plasma cf-nDNA is significant higher in early CKD (stage 1 and 2) than other stages and base plasma NGAL level is higher in advanced CKD (stage 3b, 4 and 5) than early CKD.

**Table 2:** The levels of different parameters in varying stages of CKD

	Stage 1,2 ^a^	Stage 3 ^b^	Stage 3 ^c^	Stage 4,5 ^d^	*P* value	Post hoc tests
Plasma cf-mtDNA (GE/mL)	523 ± 710	373 ± 360	420 ± 664	112 ± 134	0.209	
Plasma cf-nDNA (GE/mL)	1269 ± 1195	698 ± 527	439 ± 569	104 ± 70.01	<0.001**	a > b,c,d
Urine cf-mtDNA (GE/mL)	0.43 ± 0.69	0.98 ± 3.10	2.78 ± 10.12	6.49 ± 17.85	0.220	
Urine cf-nDNA (GE/mL)	2.83 ± 4.60	4.49 ± 12.09	15.39 ± 44.01	8.01 ± 12.08	0.351	
Plasma NGAL (ng/mL)	394 ± 175	489.6 ± 174.9	744.2 ± 272.1	1096 ± 488.7	<0.001**	a,b,<c,d
MCN (per cell)	90.7 ± 66.9	94.9 ± 77.01	92.9 ± 89.9	110.5 ± 112.7	0.901	
Urine 8-OH dG/Cr	3.73 ± 1.81	3.78 ± 2.93	4.15 ± 2.65	2.99 ± 1.53	0.416	

*P*-value by One-Way ANOVA follow with Bonferroni multiple comparisons at type I error of 0.05 level

*P <0.05, **P <0.01

Post hoc  ^a^ indicates group stage 1,2;  ^b^ indicates group stage 3a;  ^c^ indicates group stage 3b;  ^d^ indicates group stage 4,5

*MCN* mitochondrial copy number, *NGAL* neutrophil gelatinase-associated lipocalin; 8-OH dG 8-hydroxy-2-deoxyguanosine, *cf-mtDNA* cell-free mitochondrial DNA, *cf-nDNA* cell-free nuclear DNA, *GE/mL* genome equivalents/mL

### The difference of baseline parameters between different (favorable vs. unfavorable) renal outcome at 6 months (Table 3)

There are significantly lesser levels of urinary cf-mtDNA, cf-nDNA and plasma NGAL in the favorable renal outcome group. The levels of urinary 8-hydroxy-2-deoxyguanosine (8-OHdG), plasma cf-mtDNA and plasma cf-nDNA are not significantly different between favorable and unfavorable renal outcome groups.

**Table 3:** The difference of baseline parameters between favorable and unfavorable renal outcome at 6 months

	Favorable renal outcome (*n* = 53)	Unfavorable renal outcome (*n* = 70)	*P* value
Plasma cf-mtDNA (GE/mL)	441.1 ± 525.6	370.6 ± 641.9	0.538
Plasma cf-nDNA (GE/mL)	760.9 ± 732.9	554.1 ± 850.9	0.184
Urine cf-mtDNA (GE/mL)	0.489 ± 0.751	3.745 ± 12.197	0.027*
Urine cf-nDNA (GE/mL)	1.764 ± 2.388	15.119 ± 40.791	0.009*
MCN (per cell)	87.21 ± 69.40	101.55 ± 95.75	0.356
Urine 8-OH dG/Cr	3.62 ± 2.44	4.03 ± 2.44	0.346
NGAL (ng/mL)	585.24 ± 251.62	780.85 ± 391.39	0.032*

*P < 0.05, Student’s test

MCN, mitochondrial copy number; NGAL, neutrophil gelatinase-associated lipocalin; 8-OH dG, 8-hydroxy-2-deoxyguanosine; cf-mtDNA, cell-free mitochondrial DNA; cf-nDNA, cell-free nuclear DNA; GE/mL, genome equivalents /mL

Favorable renal outcome indicated eGFR at 6 months minus baseline eGFR> = 0; unfavorable renal outcome indicated eGFR at 6 months minus baseline eGFR <0

### The correlation between urinary cf-nDNA, cf-mtDNA and variables

As univariate analysis for the correlation between urinary cf-nDNA, cf-mtDNA and variables (Tables 4 and 5, Additional file 2: Figure S2 and Additional file 3: Figure S3), the urinary cf-nDNA is significant correlation with urine PCR, urine ACR, urine protein, cf-mtDNA and plasma NGAL. Concurrently, the urinary cf-mtDNA is significant correlation with urine PCR, urine ACR, urine protein and cf-mtDNA. In the multivariate analysis of predictors, we demonstrate that both urine P/C ratio and A/C ratio are the significant predictors for urinary cf-mtDNA and cf- nDNA levels (Tables 6 and 7).

**Table 4:** Univariate correlation between urinary cf-nDNA and variables

Variable	Rho correlation coefficient	*P* value
Urine A/C ratio (mg/g)	0.408	0.005*
Urine P/C ratio (mg/g)	0.314	0.007*
Urine cf-mtDNA (GE/mL)	0.448	<0.001**
Urine protein (mg/dL)	0.246	0.029*
NGAL (ng/mL)	0.322	0.001**

*P < 0.05, **P <0.01; Spearman's rho correlation

NGAL, neutrophil gelatinase-associated lipocalin (ng/mL); cf-mtDNA, cell-free mitochondrial DNA (GE/mL); GE/mL, genome equivalents/mL; urine A/C ratio, urine albumin-creatinine ratio (mg/g); urine P/C ratio, urine protein-creatinine ratio (mg/g)

**Table 5:** Univariate correlation between urinary cf-mtDNA and variables

Variable	Rho correlation coefficient	*P* value
Urine A/C ratio (mg/g)	0.375	0.008*
Urine P/C ratio (mg/g)	0.349	0.002**
Urine cf-nDNA (GE/mL)	0.448	<0.001**
Urine protein (mg/dL)	0.167	0.125
NGAL (ng/mL)	0.168	0.066

*P < 0.05, **P < 0.01; Spearman’s rho correlation

NGAL, neutrophil gelatinase-associated lipocalin (ng/mL); cf-nDNA, cell-free nuclear DNA (GE/mL); GE/mL, genome equivalents/mL; urine A/C ratio, urine albumin-creatinine ratio (mg/g); urine P/C ratio, urine protein-creatinine ratio (mg/g)

**Table 6:** Multivariate Predictors of urinary cf- nDNA

Predictors	Unstandardized Coefficients		Standardized Coefficients	p value
	B	Std. Error	Beta	
(Constant)	−14.839	24.831		0.576
NGAL	−0.017	0.024	−0.071	0.502
Urine protein	−0.144	0.150	−0.463	0.380
Urine A/C ratio	−0.074	0.019	−2.214	0.013*
Urine P/C ratio	0.085	0.014	3.526	0.002**

*R*^2^ = 0.960; *P <0.05, **P <0.01

NGAL, neutrophil gelatinase-associated lipocalin (ng/mL); cf-nDNA, cell-free nuclear DNA (GE/mL); GE/mL, genome equivalents/mL; urine A/C ratio, urine albumin-creatinine ratio (mg/g); urine P/C ratio, urine protein-creatinine ratio (mg/g)

**Table 7:** Multivariate Predictors of urinary cf- mtDNA

Predictors	Unstandardized Coefficients		Standardized Coefficients	p value
	B	Std. Error	Beta	
(Constant)	−1.790	2.295		0.471
NGAL	−0.001	0.002	−0.049	0.597
Urine protein	−0.018	0.014	−0.537	0.261
Urine A/C ratio	−0.007	0.002	−1.867	0.015*
Urine P/C ratio	0.008	0.001	3.277	0.002**

*R*^*2*^ = 0.969; *P < 0.05, **P < 0.01

NGAL, neutrophil gelatinase-associated lipocalin (ng/mL); cf-mtDNA, cell-free mitochondrial DNA (GE/mL); GE/mL, genome equivalents/mL; urine A/C ratio, urine albumin-creatinine ratio (mg/g); urine P/C ratio, urine protein-creatinine ratio (mg/g)

### Evaluation of urine cf-mtDNA, and urine cf-nDNA levels as predictors of CKD patient outcomes after 6 months

**Fig. 1:**
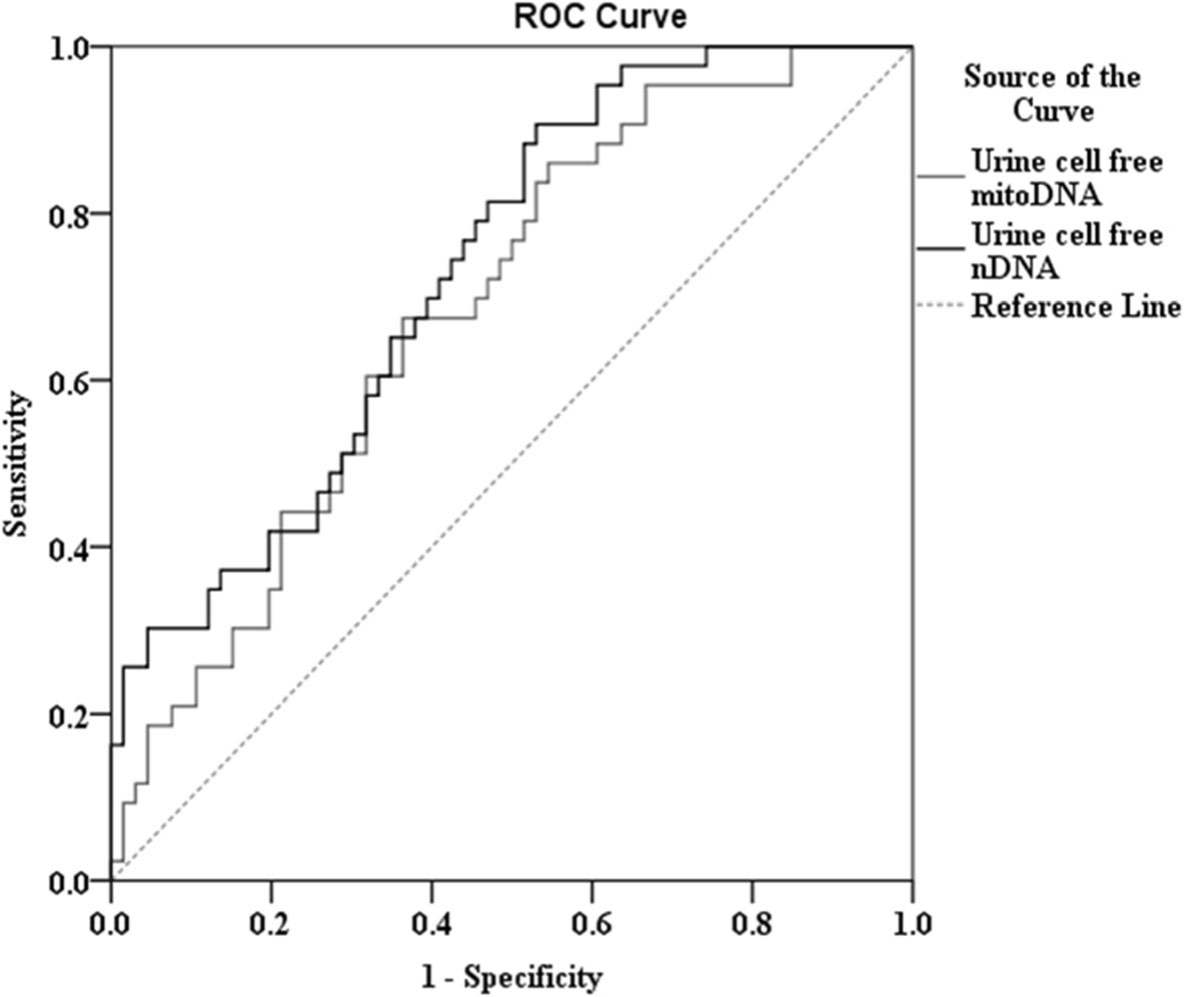
Evaluation of urinary cf-nDNA and urine cf-mtDNA as predictors of CKD patient outcomes after 6 months. The areas under the curves (AUC) were as follows: urine cf-mtDNA: 0.685 (0.586–0.784, *P* = 0.001*), and urine cf-nDNA: 0.730 (0.637–0.823, *P* < 0.001*)

Figure 1 shows the urinary cf-mtDNA and cf-nDNA levels as predictors of CKD patient outcomes after 6 months. The areas under the curves (AUC) were as follows: urine cf-mtDNA: 0.685 (0.586–0.784, *P* = 0.001*), and urine cf-nDNA: 0.730 (0.637–0.823, *P* < 0.001*). Both are better than plasma NGAL (data not shown). The optimal Youden’s index-based cut-off point was estimated. Urine cf-mtDNA cut-off value was 0.893 GE/mL, with sensitivity 0.860, 1 – specificity 0.545, and Youden’s index was 0.315. Meanwhile, urinary cf-nDNA cut-off value was 3.116 GE/mL, with sensitivity 0.907 and 1 – Specificity 0.530, and Youden’s index was 0.377. Via logistic regression analysis, we confirmed that both urine cf-mtDNA and urine cf-nDNA could be the significant predictors for dissimilar renal outcome (favorable vs. unfavorable) at 6 months (Table 8).

**Table 8:** Logistic regression analysis of predictors of favorable renal outcome at 6 months

	B	S.E.	*P* value	Odds ratio	95% C.I.	
Module 1						
Baseline eGFR	.010	.011	.377	1.010	.988	1.031
Gender (male)	.044	.494	.929	1.045	.396	2.753
Age	.023	.020	.249	1.023	0.984	1.063
NGAL	.001	.001	.152	1.001	1.000	1.003
Urine cf-mtDNA	.596	.266	.025*	1.815	1.076	3.059
Constant	−2.543	1.632	.119	.079		
Module 2						
Baseline eGFR	.027	.014	.061	1.027	.999	1.056
Gender (male)	.278	.587	.636	1.320	.417	4.175
Age	.054	.024	.027*	1.056	1.006	1.107
NGAL	.002	.001	.110	1.002	1.000	1.004
Urine cf-nDNA	.255	.102	.012*	1.290	1.057	1.575
Constant	−5.688	2.182	.009	.003		

*P < 0.05. Module 1: test urine cf-mtDNA as a covariate; Module 2: test urine cf-nDNA as a covariate

NGAL, neutrophil gelatinase-associated lipocalin (ng/mL); cf-mtDNA, cell-free mitochondrial DNA (GE/mL); cf-nDNA, cell-free nuclear DNA (GE/mL); GE/mL, genome equivalents/mL

## Discussion

To the best of our knowledge, this study is the first to show the clinical significant correlation between urine cf-mtDNA, urine cf-nDNA and divergent renal outcome at 6 months. We also propose that both urine PCR and ACR could significantly predict urinary cf-nDNA and cf- mtDNA levels.

Our study showed greater amounts of cf-nDNA in earlier stage CKD, but no correlation between urinary nuclear and mitochondria cf-DNA and CKD staging. Otherwise, there were greater quantities of urinary cf-nDNA and cf-mtDNA in the unfavorable renal outcome group. Our findings were consistent with the results from a diabetes population study that showed mtDNA was readily detectable in urinary supernatant and kidney tissue, and that its levels correlated with renal function and scarring in DN [39]. However, their study did not measure plasma cf-DNA levels or a correlation between urinary nuclear cf-DNA levels and DN prognosis.

The death of cells in the tissues is an active process that supports the homeostasis of tissues [40] and increase of cf-DNA in plasma might occur due to the enhancement of programmed cell death [41]. In AKI models, the activation of autophagy provided a significant contribution to the elimination of damaged cells from tissues [41]. Our CKD cohort findings of more plasma cf-DNA in earlier stage CKD suggested that low plasma cf-DNA in later stages, even stages 4–5, indicated the deregulation or decompensation of programmed cell death by multifactorial mechanisms. As well-known, the process of autophagy is generally assumed to be a mechanism of survival or a cytoprotective mechanism that removes damaged organelles, proteins, and other macromolecules [42, 43]. An animal study showed that kidney epithelium and podocytes were sufficient to trigger a degenerative disease of the kidney with many of the manifestations of human focal segmental glomerulosclerosis, following the prevention of autophagic flux [11].

According to the results of a number of studies, cell death accompanied by the release of cf-DNA fragments into the blood and urine can occur through autophagy as well as mechanisms of apoptosis and necrosis [42, 43]. A variety of stress stimuli can induce autophagy process, such as infection, oxidative stress, starvation, hypoxia etc. The stimulation of autophagy by these stimuli produced cellular energy stress and activated 50-adenosine monophosphate activated protein kinase (AMPK) by sensing increases in AMP:ATP and ADP:ATP ratios [43, 44]. In a DN study, Dr. Wei found a statistically significant inverse correlation between urinary supernatant and intra-renal mtDNA levels [39]. These findings were inter-related to our results where high urinary cfDNA levels might be a marker to predict kidney tissue injury in CKD patients and a worse renal outcome. According multivariate analysis, we suggested that urine P/C ratio and A/C ratio are both the significant predictors for urinary cf-nDNA and cf-mtDNA levels. However, we did not find a statistically significant correlation between urinary cfDNA and CKD staging, possibly because of the relatively small population enrolled.

The detection of microalbuminuria is a standard method to diagnose the early stages of DKD; however, some patients with microalbuminuria have advanced renal disease [45]. Microalbuminuria is not as sensitive as invasive renal biopsy. There is an unmet need to identify non-invasive biomarkers of DKD in its early stages [45–49]. Our AUC analysis showed that urinary cf-nDNA and cf-mtDNA levels were reliable to predict renal function outcomes within 6 months.

Our findings were consistent with the positive correlation between NGAL levels and CKD staging. The NGAL gene product is a protein (23–26 kDa) induced by triggers of acute kidney injury [50, 51]. NGAL is rapidly released from renal tubular cells in response to various insults to the kidney. In contrast, NGAL was recently shown to be useful in the quantitation and prediction of CKD [52, 53]. Bolignano et al. reported that NGAL closely reflected the entity of renal impairment and represented an independent risk marker for the progression of CKD [52]. Liu et al., however, demonstrated that urine NGAL levels did not predict progressive CKD [54]. NGAL is a member of the lipocalin family of proteins that has been extensively studied in acute kidney injury (AKI). NGAL is a robustly expressed protein in the kidney following ischemic or nephrotoxic injury in animals [55] and humans [56]. Via AUC analysis, we demonstrated that cfDNA might be more sensitive than well-known CKD biomarkers such as NGAL (data not shown).

There are several potential limitations to this study. First, a relatively low number of patients were investigated. Secondary, we conducted a cohort study for our clinical analysis of less than 12 months duration. Third, there was subject-to-selection bias and information on exposure was subject to observation bias from the statistic model. Only large-scale collaborative multicenter or international studies will identify important risk factors. Finally, other hard outcomes in more than 6 months, beside eGFR surrogate, could be conducted in future study.

## Conclusions

In conclusion, both urinary cf-mtDNA and cf-nDNA should be novel biomarkers to predict the prognosis of chronic renal diseases. The levels of plasma cf-nDNA and NGAL were significantly correlated with the severity of CKD.

## Supplementary information

**Additional file 1. Figure S1.** Box-whisker plots for plasma NGAL (Figure S1A) and plasma cf-nDNA (Figure S1B) levels in varying stages of CKD.

**Additional file 2. Figure S2.** Scatter-plots for correlation analysis between urinary cf-nDNA and different variables. Figure S2A Correlation between urine cf-nDNA and urine protein/creatinine ratio. Figure S2B Correlation between urine cf-nDNA and urine albumin/creatinine ratio. Figure S2C Correlation between urine cf-nDNA and urine protein ratio. Figure S2D Correlation between urine cf-nDNA and plasma NGAL. Figure S2E Correlation between urine cf-nDNA and urine cf-mtDNA.

**Additional file 3. Figure S3.** Scatter-plots for correlation analysis between urinary cf-mtDNA and different variables. Figure S3A Correlation between urine cf-mtDNA and urine albumin/creatinine ratio. Figure S3B Correlation between urine cf-mtDNA and urine protein/creatinine ratio.

## Data Availability

All data and materials are availability in the draft.

## Abbreviations

8-OH dG: 8-hydroxy-2-deoxyguanosine
ACR: albumin-creatinine ratio
AKI: acute kidney injury
cf-DNA: cell-free DNA
cf-mtDNA: cell-free mitochondrial DNA
cf-nDNA: cell-free nuclear DNA
CKD: chronic kidney disease
DNA: deoxyribonucleic acid
eGFR: estimated glomerular filtration rate
eGFR_CKD-EPI_: CKD- Epidemiology Collaboration equation
EMT: epithelial–mesenchymal transition
ESRD: end stage renal disease
GE: genome equivalents
KDIGO: Kidney Disease Improving Global Outcomes
NGAL: neutrophil gelatinase-associated lipocalin
PCR: protein-creatinine ratio
ROC: Receiver operator characteristics
