# Supplementary material for: Urinary cell-free mitochondrial and nuclear deoxyribonucleic acid correlates with the prognosis of chronic kidney diseases
Source: BMC Nephrol. 2019 Oct 28;20:391. doi: 10.1186/s12882-019-1549-x (PMC6816217; doi:10.1186/s12882-019-1549-x)

**Additional file 1: Figure S1A and S1B. Box-whisker plots for plasma NGAL and plasma cf-nDNA levels in varying stages of CKD.**

Fig.1A**.** Box-whisker plots for plasma NGAL levels in varying stages of CKD.


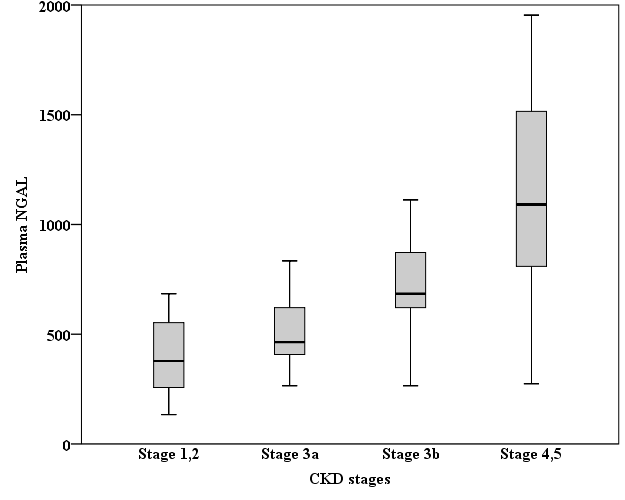


Fig.1B. Box-whisker plots for plasma cf-nDNA levels in varying stages of CKD.


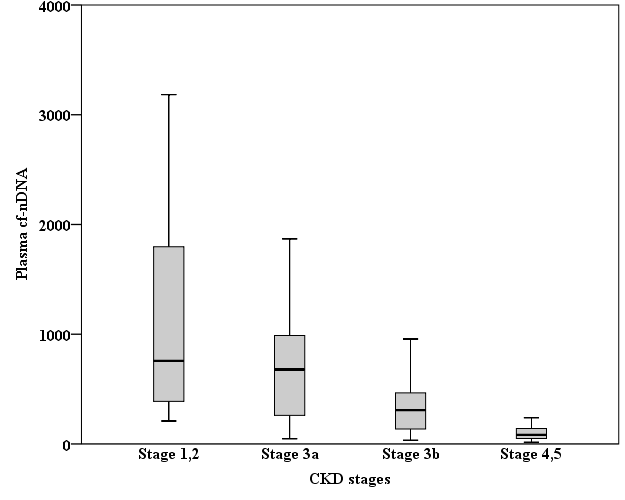

Supplement: Supplementary file 1 — Additional file 1. Figure S1. Box-whisker plots for plasma NGAL (Figure S1A) and plasma cf-nDNA (Figure S1B) levels in varying stages of CKD. [file 12882_2019_1549_MOESM1_ESM.docx]
