# Supplementary material for: Urinary cell-free mitochondrial and nuclear deoxyribonucleic acid correlates with the prognosis of chronic kidney diseases
Source: BMC Nephrol. 2019 Oct 28;20:391. doi: 10.1186/s12882-019-1549-x (PMC6816217; doi:10.1186/s12882-019-1549-x)

**Additional file 2: Figure S2. Scatter-plots for correlation analysis between urinary cf-nDNA and different variables**

2A**.** Correlation between urine cf-nDNA and urine protein/creatinine ratio


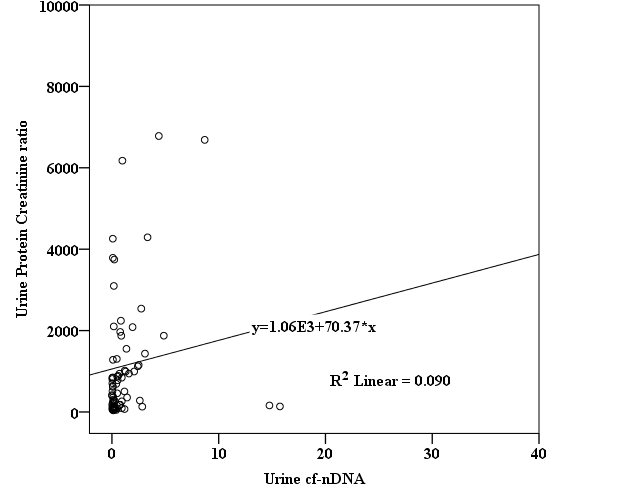


2B. Correlation between urine cf-nDNA and urine albumin/creatinine ratio


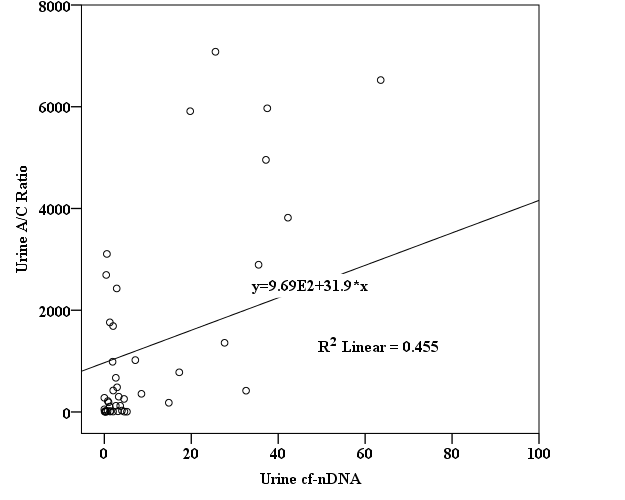


2C. Correlation between urine cf-nDNA and urine protein ratio


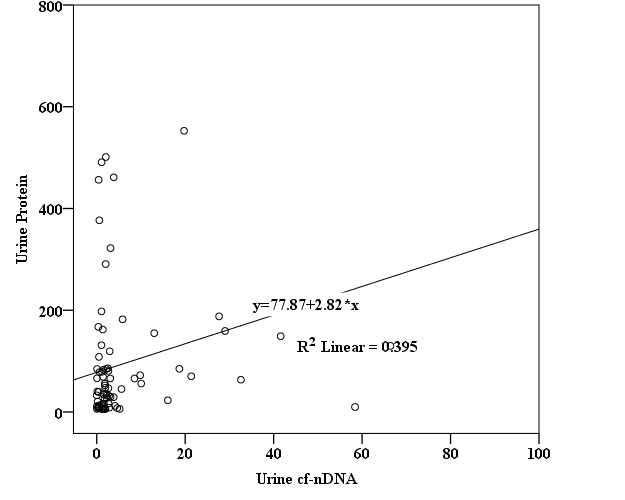


2D. Correlation between urine cf-nDNA and plasma NGAL


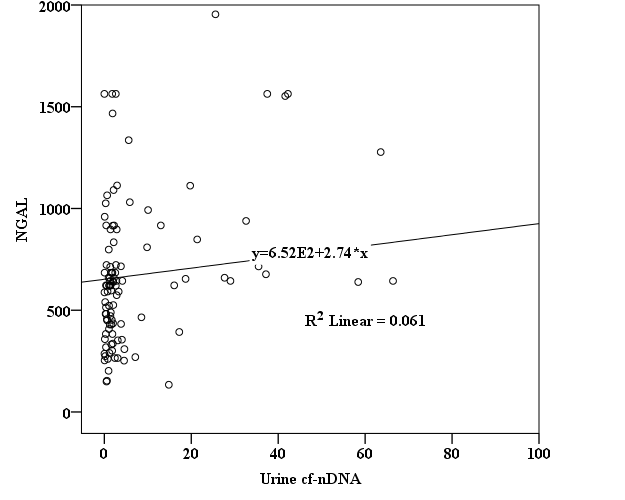


2E. Correlation between urine cf-nDNA and urine cf-mtDNA


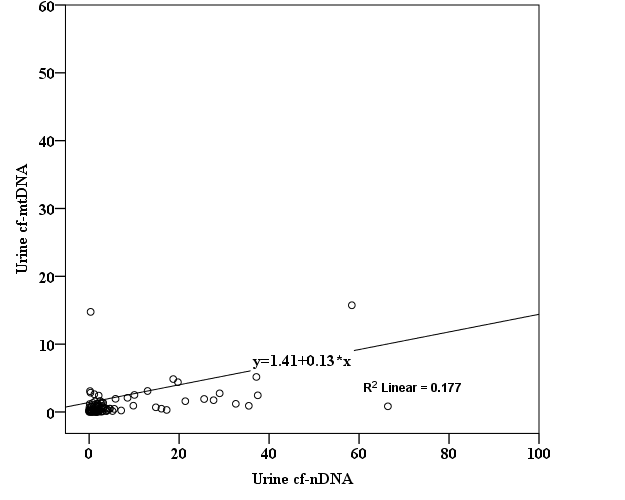

Supplement: Supplementary file 2 — Additional file 2. Figure S2. Scatter-plots for correlation analysis between urinary cf-nDNA and different variables. Figure S2A Correlation between urine cf-nDNA and urine protein/creatinine ratio. Figure S2B Correlation between urine cf-nDNA and urine albumin/creatinine ratio. Figure S2C Correlation between urine cf-nDNA and urine protein ratio. Figure S2D Correlation between urine cf-nDNA and plasma NGAL. Figure S2E Correlation between urine cf-nDNA and urine cf-mtDNA. [file 12882_2019_1549_MOESM2_ESM.docx]
