# Supplementary material for: Urinary cell-free mitochondrial and nuclear deoxyribonucleic acid correlates with the prognosis of chronic kidney diseases
Source: BMC Nephrol. 2019 Oct 28;20:391. doi: 10.1186/s12882-019-1549-x (PMC6816217; doi:10.1186/s12882-019-1549-x)
Supplement: Supplementary file 3 — Additional file 3. Figure S3. Scatter-plots for correlation analysis between urinary cf-mtDNA and different variables. Figure S3A Correlation between urine cf-mtDNA and urine albumin/creatinine ratio. Figure S3B Correlation between urine cf-mtDNA and urine protein/creatinine ratio. [file 12882_2019_1549_MOESM3_ESM.docx]

**Additional file 3: Figure S3. Scatter-plots for correlation analysis between urinary cf-mtDNA and different variables**

Fig.3A**.** Correlation between urine cf-mtDNA and urine albumin/creatinine ratio


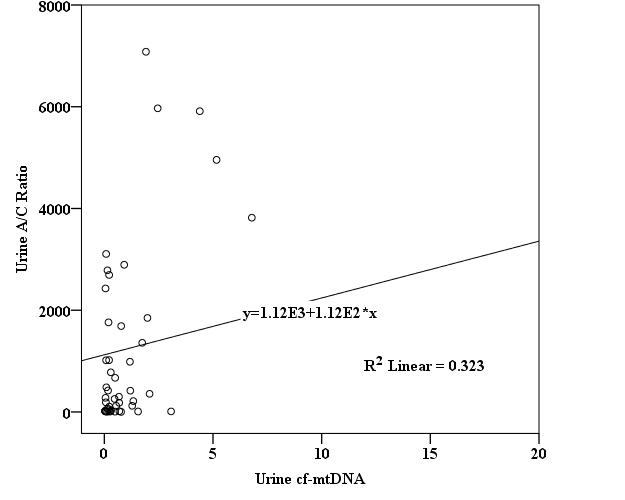


Fig.3B. Correlation between urine cf-mtDNA and urine protein/creatinine ratio

**
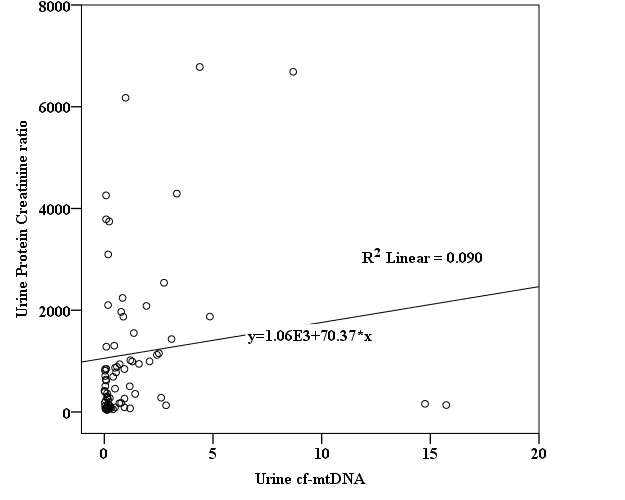
**
